# Do Perfluorinated Chemicals Enhance the Toxicity of Other Contaminants in Aquatic Organisms? A Review

**DOI:** 10.3390/toxics14050373

**Published:** 2026-04-26

**Authors:** Eliana Maira Agostini Valle, Emma Ivantsova, Maria Luisa Pracchia, Calvin Quessada Cabello, Hueder Paulo Moisés de Oliveira, Lucia Codognoto, Christopher J. Martyniuk

**Affiliations:** 1 Center for Environmental and Human Toxicology, Department of Physiological Sciences, College of Veterinary Medicine, University of Florida, Gainesville, FL 32611, USA; emavalle@unifesp.br (E.M.A.V.); eivantsova@ufl.edu (E.I.); 2 Instituto de Ciências Ambientais, Químicas e Farmacêuticas, Campus Diadema, Universidade Federal de São Paulo, Diadema 09972-270, Brazil; pracchia@unifesp.br (M.L.P.); lucia.codognoto@unifesp.br (L.C.); 3 Centro de Ciências Naturais e Humanas (CCNH), Universidade Federal do ABC, Santo André 09280-560, Brazil; calvincabello@gmail.com (C.Q.C.); hueder.paulo@ufabc.edu.br (H.P.M.d.O.); 4 Interdisciplinary Program in Biomedical Sciences Neuroscience, UF Genetics Institute, Gainesville, FL 32611, USA

**Keywords:** PFAS, pesticides, microplastics, trace metals, pollutant mixtures

## Abstract

Environmental contaminants pose threats to exposed organisms and negatively impact the nervous, cardiovascular, immune, and reproductive systems. Per- and polyfluoroalkyl substances (PFAS) are synthetic chemicals that are ubiquitous in the environment. Given that mixtures of environmental contaminants have the potential to exacerbate toxicity, we reviewed the current literature on pesticides, microplastics, or metal exposure in combination with PFAS on aquatic vertebrates and invertebrates. The objectives were to evaluate the toxicological effects of mixtures of the selected contaminants with PFAS on aquatic organisms to better understand biological responses in animals. Based on our review, data suggest that PFAS can modify the toxicity of co-occurring pollutants. For example, synergistic effects on toxicity include chlorpyrifos + perfluorohexanoic acid (PFHxA), which increased reactive oxygen species (ROS) and upregulated neurotoxicity-related genes in zebrafish, and perfluorooctanoic acid (PFOA) + atrazine, which increased the presence of malformations and oxidative stress. However, antagonistic interactions were also observed, for example, reduced herbicide toxicity in PFOA + 2,4-dichlorophenoxyacetic acid (2,4-D) mixtures. PFAS combined with microplastics often intensified oxidative stress and developmental or reproductive effects, though polyethylene microplastics attenuated perfluorooctane sulfonic acid (PFOS)-induced immunotoxicity in fish like seabass. Interactions with metals also varied, with copper and cadmium enhancing oxidative stress while mercury mixtures with PFAS showed antagonism, underscoring the complexity of mixture effects in real environments. A computational approach demonstrated that PFOS can engage in intermolecular interactions with pesticides, microplastic monomers, and metals, suggesting chemical-level effects that could modify toxicity or bioavailability. Future studies should focus on elucidating the mechanisms underlying these complex interactions, investigating effects at different trophic levels and in a broader range of species, and should consider environmentally relevant mixtures.

## 1. Introduction

The synthetic chemicals known as per- and polyfluoroalkyl substances (PFAS) were discovered in the 1930s and have since been utilized in various industries due to their advantageous properties, including their resistance to high temperatures, oil, and water [1]. Many studies to date have analyzed the biological impacts of PFAS on invertebrates and vertebrates, such as algae, fish, rodents, and humans [2,3,4]. Several PFAS, including perfluorooctane sulfonic acid (PFOS) and perfluorooctanoic acid (PFOA), are reported to bioaccumulate within species and can contribute to a wide range of biological and physiological effects (i.e., altered metabolism, endocrine disruption, oxidative stress, reproduction disruption) [5,6,7,8]. PFAS can be classified by their carbon chain lengths, whereby longer carbon chains have been associated with greater toxicity than shorter carbon chains [9]. A study evaluating the toxicities of 6 perfluorinated compounds (PFCs) with different carbon-chain lengths (4, 8, and 10) and functional groups (carboxylic and sulfonic) for microorganisms in three Chinese soils showed that chain length and functional groups influence microbial activity and adsorption processes [10]. Sulfonic PFCs exhibited greater toxicity than carboxylic ones, and toxicity increased with chain length. A similar study, using HepG2 cells to assess the toxicity of perfluorinated carboxylic acids with carbon chains ranging from 4 to 12 atoms, also demonstrated a positive correlation between chain length and cytotoxicity [11]. This in vitro study indicated that shorter chain perfluorinated carboxylic acids tend to be less toxic than PFOA. The toxicity of long-chain PFAS, such as PFOA and PFOS, has driven the search for potentially less harmful substitutes, including perfluorohexanoic acid (PFHxA), perfluorohexane sulfonic acid (PFHxS), and hexafluoropropylene oxide dimer acid (HFPO-DA, known as GenX) [12], and certain PFAS have already been phased out of production due to their toxicity. For example, PFOS began to be phased out of production in the early 2000s due to its harmful impacts on humans, which include birth defects, hormone disruption, and possible carcinogenicity [1].

PFAS’ resistance to degradation contributes to their environmental presence; however, it is also proposed that environmental concentrations of PFAS are positively correlated to population density [13]. PFAS have been found to range from varying concentrations (ng/L–µg/L) in aquatic ecosystems and have been reported in ng/g in various organisms, including humans, fish, and rodents. For instance, Hoff et al. [14] measured the concentrations of PFOS and organohalogen pollutants in the liver of gibel carp (*Carassius auratus gibelio*), carp (*Cyprinus carpio*), and eel (*Anguilla anguilla*) collected from Belgium, and PFOS was found to range from 11.2 to 781, 11.3 to 1822, and 17.3 to 9031 ng/g wet weight in the species, respectively. These chemicals have already been found in different fish species, such as carp, sea bass and tilapia, and the data suggests different toxicity targets (such as the heart, liver, immunity and reproductive system) [15]. In aquatic species, perfluorohexanoic acid (PFHxA) and perfluorohexanesulfonic acid (PFHxS) exert toxicity resulting in oxidative stress and endocrine disruption [16]. Currently, there are hundreds of PFAS in the environment with little data on their toxicity.

Due to the growing global concern over the persistence, mobility, and toxicity of per- and polyfluoroalkyl substances (PFAS), regulatory actions targeting these compounds have intensified across different regions of the world. In the United States, the Environmental Protection Agency (EPA) established in April 2024, through the National Primary Drinking Water Regulation (NPDWR), a maximum contaminant level of 4 ppt for PFOS and PFOA in drinking water, with full implementation expected by 2029 [17]. Also in 2024, PFAS were officially designated as hazardous substances under the Comprehensive Environmental Response, Compensation, and Liability Act (CERCLA), expanding legal liability for contaminated sites by including these compounds in the list of substances subject to remediation and environmental accountability [18]. In parallel, the Food and Drug Administration (FDA) consolidated, between 2020 and 2024, the voluntary and progressive phase-out of PFAS in food packaging [19], while state-level legislation has expanded prohibitions on the use of these compounds in consumer products, such as in California [20] and in Maine, where Law No. 38 M.R.S. §1614 establishes progressive restrictions on PFAS use through 2030 [21]. In the European Union, regulations are more rigorous and specific. The REACH Regulation (Regulation (EC) No 1907/2006, in force since 2007) establishes the criteria for identifying substances of very high concern. The European Union’s Drinking Water Directive, scheduled for implementation in January 2026, introduced mandatory PFAS monitoring for all member states [22]. In addition, the Toy Safety Regulation, in force since January 2026, prohibits the use of PFAS in toys [23]. In France, Decree No. 2025-1376, issued on 28 December 2025, restricts the manufacture and marketing of textiles, waxes, cosmetics, footwear, and waterproofing products containing PFAS, and establishes specific residual limits of 25 ppb for individual PFAS (excluding polymers), 250 ppb for the sum of PFAS by targeted analysis, and 50 ppm for total PFAS (including polymers) [24]. There is a clear regulatory movement toward limiting and progressively phasing out these compounds, which poses significant challenges for industries in the search for substitutes [25]. However, replacing PFAS is complex and requires careful case-by-case evaluation, considering the specific application, the compound’s function in the formulation, and the compatibility and stability of potential alternatives in the final product. This individualized assessment reflects not only differences in physicochemical properties among compounds but also regulatory and safety requirements, which demand substitutes with lower environmental and toxicological impact without compromising process or product performance.

Similarly, many pesticides have been banned or phased out of production due to their toxicity on non-target organisms. Pesticides are utilized worldwide to increase the harvest yield in the agricultural sector and to mitigate pests in residential areas; thus, thousands to millions of kilograms of herbicidal products can be applied yearly. For example, about 240 million pounds (108.86 × 10^6^ kg) of glyphosate were reportedly applied by ranchers and farmers in 2014 [26]. Many herbicides in the environment are persistent due to their resistance to degradation, which can compromise soil health and cause water contamination. Consequently, numerous studies to date have assessed the impacts of pesticide application to non-target organisms. For example, juvenile *Labeo rohita* fish exposed to bifenthrin and chlorpyrifos revealed DNA damage and genotoxic effects [27]. Additionally, organophosphate insecticides have been found to have pronounced effects on fish cholinesterase (ChEs) followed by carbamates > organochlorines and pyrethroids, where the herbicides organophosphates and oxazolidinones were noted to decrease the average activity of ChEs [28]. As another example, Barbosa et al. [29] detected 0.33 mg/kg of azoxystrobin, 0.033 mg/kg of epoxiconazole, and 0.017 mg/kg of chlorpyrifos in tambaqui fish muscle tissue samples collected in Rondonia State, Brazil, with values above those permitted by the Brazilian legislation. Indiscriminate pesticide use continues to be a persistent issue globally. Additionally, a study investigated the synergistic neurotoxic effects of PFOS and glyphosate using SH-SY5Y neuronal cells and C6 astrocytic cells, demonstrating that co-exposure at individually non-toxic concentrations intensified oxidative stress and neuroinflammatory responses [30]. These findings reinforce the importance of considering synergistic interactions between pollutants when assessing neurotoxic risks.

Another pressing global issue is that of micro- and nanoplastics and their toxicity to aquatic wildlife. Substantial portions of plastic waste are scattered throughout our environment. According to the United States Environmental Protection Agency [31], plastic particles that range from 5 mm (mm) to 1 nanometer (nm) in size are known as microplastics (MPs). MPs can be categorized as primary or secondary MPs. Primary MPs are purposefully manufactured to be small to be incorporated in consumer products, whereas secondary MPs result from the degradation of larger plastic particles. MPs can penetrate organisms leading to their accumulation and potential toxicity mechanisms. For example, microplastics have been reported to bioaccumulate in zebrafish organs following exposure, causing toxic effects like structural deterioration of the gonads, increased oxidative stress, developmental disorders, incomplete functioning of the digestive system, and decreased locomotor activity [32]. MPs can sorb PFAS and act as long-distance transport vectors in aquatic environments. Furthermore, atmospheric deposition of PFAS and MPs has been reported in both urban and rural areas [33]. These processes increase the potential for combined toxicity, posing risks to ecosystems and human health. Co-exposure to MPs and PFAS is often associated with altered PFAS partitioning and increased uptake by organisms, with reports of higher bioaccumulation compared to exposure to PFAS alone. These effects are frequently accompanied by intensified oxidative stress, immune dysregulation, metabolic disturbances, and reproductive impairment, particularly in aquatic invertebrates and early life stages of fish [34].

Like PFAS and MPs, trace metals (i.e., cadmium (Cd), copper (Cu), lead (Pb), mercury (Hg), nickel (Ni), and zinc (Zn)) also have the potential to accumulate within organisms, which can lead to chronic poisoning [35]. Exposure to such metals in various fish species (i.e., goldfish (*Carassius auratus*), rainbow trout (*Oncorhynchus mykiss*), common carp (*Cyprinus carpio*)) has been found to contribute to oxidative stress through excessive ROS production [36,37,38], and damage tissues, such as the intestines, liver, and gills [37,39], which can induce DNA damage/modification [37,38,40].

Despite a wealth of knowledge regarding the individual effects of chemicals on biological systems, their combined sub-lethal toxicity effects are not well studied. Mixture toxicity will influence the dosage and potency of individual compounds; however, the interaction of these compounds can also have antagonistic or synergistic effects [41]. The aim of this review was to compile toxicity data for co-exposures to PFAS with either pesticides or metals or microplastics. Furthermore, there is growing concern regarding the bioaccumulation of these species in fish muscles, as this is an important nutritional source [42]. Given the widespread utilization and persistence of PFAS, we surveyed the available studies from the literature examining the effects of PFAS in combination with pesticides, MPs, or trace metals in vertebrates and invertebrates. As a result, a more accurate representation of the environmental and health risks related to these compounds may be obtained by examining cumulative exposures rather than individual ones.

## 2. Literature Review and Methodology

**Figure 1:**
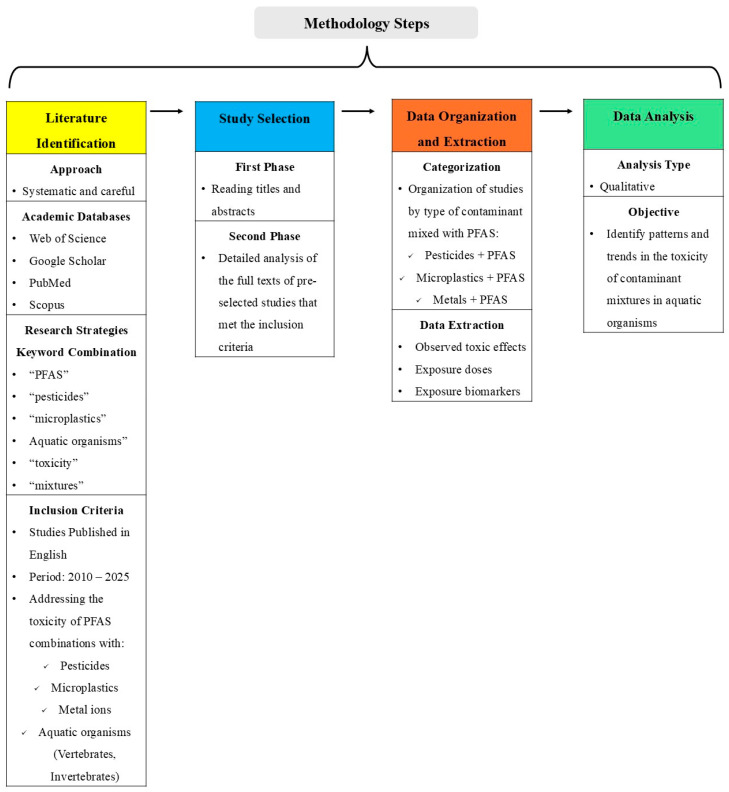
Overview of the methodology for the literature review, including literature identification, study selection, data organization and extraction, and data analysis of PFAS-containing contaminant mixtures in aquatic organisms.

We focused on aquatic organisms such as fish, algae, and microorganisms at different endpoints. We collected and analyzed the available literature based on the recognized academic databases, such as PubMed, Scopus, Web of Science, and Google Scholar, using search strategies combining related keywords, such as: “PFAS”, “pesticides”, “microplastics”, “metal ions”, “aquatic organisms”, “toxicity”, and “mixtures”, among others. After this first screening, specific contaminants were used for the search, such as “PFOS and Pesticides and fish” or “PFAS and glyphosate and toxicity”, to expand the search. To ensure comprehensive searches, the most well-studied PFAS, pesticides, and metal ions were also used (i.e., PFOA, PFOS, PFOSA, glyphosate, chlorpyrifos, Cu, iron (Fe), etc.). Searches were conducted between May 2024 to May 2025. Studies were published in English, between 2010 and 2025, addressing the toxicity of combinations of PFAS with either pesticides, microplastics or metal ions on aquatic organisms (both vertebrates and invertebrates). The inclusion criteria were: (1) the study involved an exposure experiment with at least one PFAS and at least one pesticide, microplastic, or metal, in environmental samples (water/sediment), and (2) measured at least one biological outcome in an aquatic species. Our methodology steps are portrayed in Figure 1.

Methods for chemical interactions: ground state geometry optimization for the molecules was performed by density functional theory (DFT) [43] using the B3LYP functional with the GD3 term for Grimme’s dispersion correction [44] and the 6-311G(d,p) basis set [45]. In the optimization procedure, the solvent effect was also introduced using the polarizable continuous model of integral equation formalism (IEFPCM) [46], with water as the solvent. First, the separated molecules had their geometry optimized, and then, they were combined with PFOS in different positions, based on their MEP and potential h-bond interactions. Then, they were optimized to see their interactions with the PFAS species. Calculations were performed with the Gaussian 09 program package [47].

## 3. Perfluorinated Chemicals and Co-Exposures

### 3.1. PFAS and Pesticides: Co-Occurrence and Toxicity

Globally, millions of tons of pesticides are used annually for agricultural purposes, within which about 3.70 million tons of pesticides were applied in 2022 alone [48]. Pesticides can accumulate in the environment and organisms from runoff, aerial applications, and improper disposal [49]. Additionally, pesticides may contaminate aquatic environments, potentially causing toxic effects on non-target organisms, which may contribute to declines in biodiversity over time [50]. The studies on mixtures of PFAS and pesticides described in this review encompass compounds from different chemical classes and with distinct biological targets, including herbicides such as atrazine, 2,4-D, diuron, and paraquat, the insecticide chlorpyrifos, and fungicides such as cyproconazole and triadimefon. These pesticides belong to varied chemical structural groups such as triazines, triazoles, bipyridiniums, and organophosphates, which may contribute to the diversity of effects observed with PFAS. Despite evidence of environmental accumulation of PFAS and pesticides, limited studies evaluate the impact of these compounds simultaneously.

#### 3.1.1. Co-Occurrence of PFAS and Pesticides in Aquatic Species

The coexistence of PFAS and pesticides has been reported in environmental matrices (i.e., surface water, sediment, vegetation) in Saudia Arabia [51]. In addition to 12 organophosphates and 34 pharmaceutical and personal care products (PPCPs), 64 pesticides and 21 PFAS were detected in the samples analyzed. The median concentrations of PFAS in water, sediment, vegetation for human consumption, agricultural crops, and natural vegetation were 29.7 ng/L, 5.66 ng/g, 0.46 ng/g, 3.2 ng/g, and 1.88 ng/g, respectively, while pesticides had median concentrations of 231 ng/L, 40.4 ng/g, 42.0 ng/g, 57.5 ng/g, and 10.3 ng/g, respectively [51]. Additionally, in an assessment of sediments collected from a sheltered bay on the west coast of Norway that was used as waste disposal, Dale et al. [52] reports up to 26 μg/kg polychlorinated biphenyls (PCBs), which exceeds the environmental quality standards established by European Union legislation. PFOS, PFOA and organochlorine pesticides, including dichlorodiphenyltrichloroethane (DDT) and 2,4-dichlorophenoxyacetic acid (2,4-D), were also detected in all the sediments analyzed.

Various studies report on the coexistence of these two chemicals within fish. Atlantic cod (*Gadus morhua*) were exposed for six weeks in a bay in Norway used as a waste disposal with a known presence of PFAS and pesticides [52]. A total of 15 PFAS and five organochlorine pesticides from the DDT family were analyzed, and several of them were detected in cod liver at concentrations in the ng/g range. The Hepatosomatic Index and Condition Factor were significantly lower in cod kept in cages near the waste disposal site, suggesting a compromised state of health. In addition, protein biomarkers such as vitellogenin, metallothionein, and oxidative stress enzymes were measured, with significant reductions in catalase (CAT) and glutathione S-transferase (GST) activities in the liver, indicating oxidative stress. Gene expression also showed an increase in the transcriptions of genes related to lipid metabolism and steroidogenic enzymes, suggesting possible impacts on the reproductive system. Finally, high levels of 17β-estradiol (E2) were observed in the plasma of cod from the innermost season, pointing to possible endocrine dysregulation [52]. However, with such field studies, it is not possible to attribute these biological responses to any one class of chemical.

Other studies have measured PFAS and pesticides in the same tissues of fish. For example, tissues of aquatic species from The Netherlands, including salmon (*Salmo salar*), pangasius (*Pangasius hypophthalmus*), tilapia (*Oreochromis mossambicus*, *Oreochromis niloticus*), trout (*Oncorhynchus mykiss*, *Salmo trutta*), and shrimp (*Penaeus monodon*, *Penaeus vannamei*, *Litopenaeus vannamei*), were analyzed by Van Leeuwen et al. [53]. Samples revealed variations in the degree of contamination between species, with salmon being the most contaminated species, followed by trout, tilapia, pangasius, and shrimp. Salmon, which has the highest lipid content, accumulated the highest concentrations of polychlorinated biphenyls (PCBs), the pesticide DDT, and PFOS, with 10,860 pg/g wet weight for PCBs, more than 1 ng/g for DDT and up to 600 pg/g for PFOS. Species with plant-based diets, such as tilapia and pangasius, tended to accumulate fewer contaminants, which reflects the influence of diet and lipid content on the bioaccumulation of these compounds. Among the organochlorine pesticides (OCPs), concentrations varied according to species, but all were below the maximum limits allowed by European Union legislation. Tilapia and pangasius showed lower levels, while salmon showed significantly higher concentrations. In relation to PFAS, PFOS was the most prevalent contaminant in shrimp, while the other PFAS samples were at low or non-detectable levels [53]. Lastly, the presence of perfluorooctane sulfonic acid (PFOS) was assessed in liver samples of gibel carp (*Carassius auratus gibelio*), carp (*Cyprinus carpio*), and eel (*Anguilla anguilla*) collected in the Flanders region of Belgium. The same study also investigated the presence of 13 organochlorine pesticides, as well as other organohalogenated compounds. Among the pesticides detected, *p,p′*-dichlorodiphenylethylene (*p,p′*-DDE) and *p,p′*-dichlorodiphenyldichloroethane (*p,p′*-DDD) were identified, with mean concentrations of 14.6 ng/g and 13.8 ng/g of *p,p′*-DDE, and 6.2 ng/g and 4.8 ng/g of *p,p′*-DDD in carp and eel, respectively. Serological alterations observed in eel liver were attributed to the presence of PFOS, with no apparent contribution from the detected pesticides, suggesting that other organohalogenated compounds present in the samples may have contributed to the observed effects [14]. Nevertheless, it is difficult to definitively conclude that the presence of PFOS exacerbated toxicity in natural populations of fish.

#### 3.1.2. Combined Toxicity of PFAS and Pesticides in Aquatic Species

Few laboratory studies evaluate the combined toxicity of PFAS and pesticides. One study exposed zebrafish (*Danio rerio*) from 5 to 800 mg/L PFOA and 5 to 15 mg/L atrazine for 5 days to determine if co-exposure contributed to stronger compound toxicity [54]. Co-exposure resulted in more than a two-fold higher incidence of malformations (i.e., yolk sac abnormalities, liver abnormalities, spinal curvature) in a dose-dependent manner compared to single-compound exposure. Additionally, co-exposure significantly inhibited embryo hatchability, whereas single exposure to atrazine did not significantly affect hatching rate. Amino acid metabolism was analyzed, and more complex alterations were recorded in the co-exposure, including cysteine, methionine, and serine being more activated. Regarding reactive oxygen species (ROS), ROS were significantly increased by 56.5% in co-exposure compared to 29.3% and 38.6% in the single PFOA and atrazine exposure, respectively. Overall, combined exposure exerted stronger toxicity. In another study conducted by Zoupa et al. [41], zebrafish were exposed to up to 300 µM cyproconazole or triadimefon, two fungicides, with up to 30 µM PFOS over a course of 5 days. The maximum response in fish treated with PFOS and triadimefon was higher than when combined with cyproconazole; however, this variation was proposed to be due to the lethality observed with PFOS. In the study by Valle et al. [55], developing zebrafish were exposed to 0.62/10, 6.2/10, 62/10, and 620/10 μg/L chlorpyrifos/PFHxA for 7 days. At the highest concentration, survival and locomotor activity were reduced significantly. Additionally, co-exposure caused genes associated with neurotoxicity and oxidative stress to be upregulated and ROS levels were reduced at lower chlorpyrifos concentrations, indicating a potential modulatory interaction on oxidative stress pathways.

Other mixture experiments have been conducted in the laboratory. Bizarro et al. [56] exposed Atlantic cod to a mixture containing PFOA, chlorpyrifos, bis-(2-ethylhexyl)-phthalate (DEHP), and 17α-ethinylestradiol (EE2) for 48 h. The lowest concentration mixture used contained 0.01 µM EE2 and 0.1 µM chlorpyrifos, DEHP, and PFOA. The other mixture tested included environmentally relevant concentrations (0.01 µM EE2, 0.1 µM chlorpyrifos, and 1 µM DEHP, and PFOA). Significant effects on liver metabolism were detected following exposure to both mixtures, in which transcripts related to lipid/cholesterol metabolism (*fabp* and *hmg-CoA*), vitamin D metabolism (*cyp24a1*), and xenobiotic metabolism (*cyp3a*) were altered only in mixture scenarios. It should be noted that DEHP is a plasticizer and EE2 is a pharmaceutical estrogen, which may have contributed to the biological impacts recorded.

The experiments conducted by Rodea-Palomares et al. [57] with recombinant bioluminescent cyanobacterium Anabaena CPB4337 analyzed the interaction between PFOA and PFOS with 2,4-dichlorophenoxyacetic acid (2,4-D). The results obtained were based on the inhibition of constitutive luminescence caused by the presence of any toxic substance. Binary combinations of PFOA with 2,4-D showed antagonistic behavior throughout the range of effects analyzed. In contrast, combinations of PFOS with 2,4-D showed synergistic interactions over most of the effect range. These variations in the behavior of the mixtures may be related to the relative hydrophobicity of the compounds, since PFOA and PFOS have different cell permeability properties, affecting their interactions with the compounds. Lastly, Rodea-Palomares et al. [58] pre-exposed cyanobacterium Anabaena CPB4337 cells to 5 mg/L PFOA or PFOS for 72 h before being exposed to one of four herbicides: 2,4-D, atrazine, diuron, or paraquat. Cells were pre-exposed to PFAS to determine their impact on cell toxicity, and both PFAS were found to significantly alter herbicide toxicity. PFOA pre-exposure significantly increased the toxicity of all herbicides except for atrazine. For example, the toxicity of paraquat on cells doubled with PFOA pre-exposure. Regarding PFOS pre-exposure, toxicity of paraquat and diuron increased, while the toxicity of atrazine decreased.

Overall, limited data are available to examine the potential biological and physiological consequences of PFAS and pesticides in mixture. Additionally, to our knowledge, no studies to date have examined the impact of both PFAS and pesticides on mammalian models. Considering the synergistic effect reported in aquatic organisms exposed to these chemical classes, further research on mammalian data is worthy of completion. Table 1 summarizes studies of the toxicity of PFAS and pesticides in different aquatic organisms.

### 3.2. PFAS and Microplastics: Co-Occurrence and Toxicity

Plastic production continues to increase annually. According to the United Nations Environment Program [59], about 400 million tons of plastic is produced annually. Additionally, it is estimated that 9–14 million tons of plastic entered aquatic ecosystems annually in 2016; however, this range is projected to increase to 23–27 million tons by 2040. Overall, the projected increase in plastic production is associated with plastic accumulation in aquatic ecosystems. Over time, plastic degrades into MPs, which allow for easy ingestion or absorption due to their small size. Various studies have elaborated on the impacts of MP presence in aquatic organisms (Table 1) [67,68,69].

#### 3.2.1. Co-Occurrence of PFAS and Microplastics in Aquatic Species

Very few studies elaborate on the co-occurrence of PFAS and microplastics in fish tissue and water. In terms of fish, Espinosa-Ruiz et al. [61] analyzed accumulation of PFOS and polyethylene MPs (PE-MPs) in European seabass (*Dicentrarchus labrax* L.) livers after being fed 100 mg/kg PE, 4.83 μg/kg PFOS, or a combination of the two compounds (4.83 μg/kg PFOS + 100 mg/kg PE) for 21 days. No accumulation was noted in the fish fed PE or PE-PFOS; however, bioaccumulation data completed by Islam et al. [62] contradict this data as combined exposure to PE-MPs and PFOS enhanced PFOS accumulation in all soft tissues of clams (*Scrobicularia plana*) after being exposed for 14 days to PE-MPs (1 mg/L) and PFOS (55.7 µg/g or 46.1 µg/g). Cocci et al. [70] described the presence and effects of MP and PFAS accumulation in the gastrointestinal tract and coelomic fluid of sea cucumber (*Holothuria tubulosa*) specimens. The types of polymers found were mainly polyethylene (PE) and polypropylene (PP). Seven PFAS were also detected, including PFBS, PFHpA, PFOA, and PFHxA. In this study, however, no correlation was observed between PFAS and MP bioaccumulation.

#### 3.2.2. Combined Toxicity of PFAS and Microplastics in Aquatic Species

Regarding combined toxicity of PFAS and microplastics on aquatic organisms, Espinosa-Ruiz et al. [61] found that immunological effects by PFOS are reduced in European seabass (*Dicentrarchus labrax* L.) when fed a diet with PFOS and polyethylene MPs (PE-MPs). Fish were fed 100 mg/kg PE, 4.83 μg/kg PFOS, or a combination of the two compounds (4.83 μg/kg PFOS + 100 mg/kg PE) for 21 days. Several immune and stress-related genes were also analyzed, and fish fed the PE-PFOS diet had decreased their regulation compared to the single-compound diets. Additionally, the combined diet increased bactericidal activity by 93%. Principal component analysis was used to examine the relationships between the parameters assessed, and it was found that seabass fed PE-PFOS clustered closer to those fed PE alone than when data were grouped by treatment group, confirming that PE lessens the impact of PFOS. The study by Islam et al. [62] noted increased superoxide dismutase (SOD) activity of around 1.7 and 1.8 times (*p* < 0.05) in clam gills and digestive glands after a 14-day exposure to PE-MPs (1 mg/L) and PFOS (55.7 µg/g or 46.1 µg/g). In algae, Zhao et al. [63] examined single or co-exposure of PFOA (0.05, 0.5, 5 mg/L) and polystyrene MPs (PS-MPs; 10 mg/L) for 96 h in the microalga *Chlorella sorokiniana,* where it was reported that co-exposure induced greater biotoxicity. The half maximal effective concentration (EC_50_) was significantly reduced in co-exposures compared to other treatments and cell morphology (i.e., cell membrane and wall destruction) was more severely impacted with co-exposure. The ROS ratio was 209.35% following co-exposure compared to 160.01% following single exposure to PFOA. Additionally, photosynthetic inhibition of cells was also ranked as the following: PS-MPs + PFOA > PS-MPs > PFOA. The effects of co-exposure to polyvinyl chloride (PVC) and PFOA on the algae *Microcystis aeruginosa* for 15 days at 50.0 mg/L PVC + 100.0 ng/L, 10.0 μg/L, 1.0 mg/L, 20.0 mg/L, and 100.0 mg/L PFOA promoted the synthesis and release of Microcystin-LR, had a synergistic effect on inhibiting algal growth, and antagonistic effects on CAT activity and malondialdehyde (MDA) content were observed [64]. Lastly, Soltanighias et al. [66] exposed *D. magna* to chronic exposures of 70 ng/L PFOS, 7 ng/L PFOA, and 50 mg/L polyethylene terephthalate (PET). Mixtures of two or all three variables were tested, and the exposure continued until each *Daphnia* had released its second brood. Combined toxicity was more severe with joint exposure than single exposure, in which about 59% of the effect was additive and 41% synergistic. Joint exposure also delayed sexual maturity, suppressed reproduction, triggered developmental failures, and reduced somatic growth. These studies indicate that the presence of microplastics and PFAS should concern the environment and, consequently, human health. In this sense, studies that evaluate the toxicity of these compounds in different environments and conditions are increasingly necessary.

### 3.3. PFAS and Metals: Co-Occurrence and Toxicity

Metal compounds can be introduced into the environment in various ways, such as by agricultural irrigation, fertilizer consumption, pharmaceutical production, and soil erosion [71]. Additionally, environmental levels of metals are increasing due to industrialization and urbanization [72], and, though treatment methods to reduce levels in the environment have been created, many are costly and time-consuming. Trace metal exposure has been noted to have consequences for both humans and animals, including disruptions to the gastrointestinal, reproductive, respiratory, and nervous systems [73,74]. Additionally, some trace metals can transform into a more toxic form. For example, Hg can transform into methylmercury (MeHg), which can be readily absorbed by organisms and biomagnified in the food chain [75]. The assessment of Cu(II), Cd(II), and Fe(II) influence on the adsorption of PFOA, PFOS, PFDA, and GenX onto goethite (α-FeOOH) showed that the presence of metals significantly alters the behaviors of these PFAS. Cu(II) and Cd(II) enhanced the adsorption of PFOS and PFDA through ternary complex formation, while slightly reducing the adsorption of PFOA and GenX. Fe(II), in turn, markedly increased the adsorption of all PFAS, exhibiting higher reactivity than the other cations [76].

#### 3.3.1. Co-Occurrence of PFAS and Metals in Aquatic Species

Both metals and PFAS are present in the environment and can bioaccumulate in different aquatic organisms. The co-occurrence of each type of pollutant in tissues of aquatic species has been documented. An analysis carried out on a deceased and pregnant adult female bluntnose sixgill shark (*Hexanchus griseus*) recovered in Coles Bay, Vancouver Island in 2019, detected the presence of perfluorinated compounds (such as PFOS, PFUnDA, and PFTeDA) and metal ions (such as Cr, Cd, Cu, and Pb) in the liver of the mother and offspring [77]. The study shows that these chemicals are transferred from mother to offspring, with PFAS being deposited alongside metals. In another study conducted in Haizhou Bay, eastern China, the presence of metal ions and PFAS in the local biota was observed following analysis of various marine organisms including fish, shrimp, and gastropods [78]. Among the metals, Zn was the most abundant, followed by Cu and chromium (Cr), while Cd and Hg were present at relatively low levels. Twelve PFAS were detected, with PFOS found in over 50% of the samples, followed by perfluorooctanoic acid (PFOA). Although some compounds were detected at concentrations considered safe, limited to human consumption, their co-occurrence highlights the need for further studies on the combined effects of these contaminants on aquatic species.

#### 3.3.2. Combined Toxicity of PFAS and Metals in Aquatic Species

Metal ions occur naturally in the environment, such as Cu and Fe, but can also result from anthropogenic sources, particularly industrial discharges, as is the case with Pb and Cd. Likewise, PFAS compounds have been increasingly reported in environmental matrices, as previously discussed. Given the frequent co-occurrence of these contaminants in aquatic ecosystems, recent studies have focused on evaluating their combined toxicological effects. Some toxicity studies have assessed the interactions and impacts caused by transition metal dichalcogenides (TMDCs—molybdenum disulfide (MoS_2_) and tungsten disulfide (WS_2_)), and seven PFAS in cell lines and zebrafish. The studies suggested that TMDCs-PFAS interactions in aqueous media significantly increased the bioaccumulation of the species in zebrafish, which consequently increased oxidative stress in the liver and intestines of zebrafish, demonstrated by increased levels of ROS and other enzymatic activities [79]. In goldfish, exposure to PFOA (1.21 and 12.10 µmol/L), PFOS (1 and 10 µmol/L), Cu (0.79 and 3.15 µmol/L), PFOA + Cu (1.21 + 0.79 µmol/L and 12.10 + 3.15 µmol/L), or PFOS + Cu (1 + 0.79 µmol/L and 10 + 3.15 µmol/L) for 4 days caused significant inhibition of antioxidant defense enzymes, such as CAT and SOD enzyme activities, with mixtures compared to single exposures [60]. Toxicity of the treatment groups were also ranked: PFOA < PFOS < Cu < PFOA + Cu < PFOS + Cu. Thus, in this study, combined exposure of PFOS with a metal led to higher levels of toxicity in the goldfish.

The effect of Cd and PFOS exposure on aquatic oligochaete *Limnodrilus hoffmeisteri* at various pH values was investigated, and it was observed that pH values interfere with exposure impacts where combined Cd/PFOS exposure may increase acute toxicity but decrease internal concentrations of Cd [65]. Lastly, cyanobacterium Anabaena CPB4337 exposed to binary and tertiary mixtures containing PFOA or PFOS with Hg and Cd were examined by Rodea-Palomares et al. [57]. Regarding PFOA mixtures, a combined exposure of PFOA and Hg showed strong antagonism where PFOA, Hg, and Cd led to dual synergistic/antagonistic behavior. Regarding PFOS mixtures, both the binary and tertiary mixtures showed strong antagonism. Such interaction towards trace metals may be due to the ability of PFAS to stabilize these metals through complexation or ion exchange. A study investigated the response of *Vallisneria natans* (*V. natans*) to combined exposure to PFOS and Cu at concentrations of 1.0, 10.0, and 100.0 μg/L [80]. The results indicated that both individual and combined treatments triggered the metabolism of ROS, as evidenced by increased activities of glutathione (GSH), MDA, CAT, total superoxide dismutase (TSOD), and an overall antagonistic joint toxicity. Transcriptomic analyses further revealed that the expression levels of detoxification-related genes, such as peroxidase (PER), glutaredoxin 9 (GRX9), and glutathione S-transferase 3 (GST3) were upregulated in response to exposure. Moreover, both single and combined exposures induced the upregulation of transporter-related genes encoding aquaporins, rapid-type anion channels, and P-type ATPase copper exporters, thereby enhancing the uptake of PFOS and Cu in *V. natans* and promoting detoxification processes. This study confirmed that the individual toxicity of PFOS and Cu was greater than that observed under co-exposure conditions, suggesting an antagonistic interaction between PFOS and Cu in submerged macrophytes.

### 3.4. Theoretical Calculations

**Figure 2:**
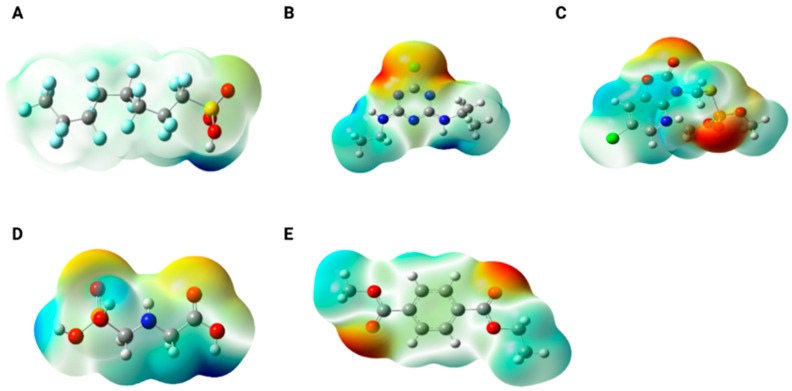
Electrostatic potential map for the compounds of (**A**) PFOS, (**B**) atrazine, (**C**) azamethiphos, (**D**) glyphosate, and (**E**) PET monomer. Regions in red depict high density of negative charge and regions in blue depict high density of positive charge.

We also performed theoretical calculations, using PFOS as a model compound, to determine whether this molecule could interact with common environmental contaminants such as pesticides, microplastics, and metals. For this purpose, three herbicides from different chemical classes were selected: atrazine, a triazine herbicide; azamethiphos, and glyphosate, both organophosphorus compounds with distinct structures. In addition, a PET monomer was included to represent a microplastic particle. Figure 2 shows the electrostatic potential maps of the molecules studied. As observed, PFOS (Figure 2A) exhibits a slight negative charge density over the oxygen atoms, while the remainder of the chain behaves as a traditional alkyl chain, displaying overall neutrality. This neutrality arises from the cancelation of the C–F bond polarity vectors. Atrazine (Figure 2B) presents a high negative charge density around the chlorine atom, whereas the amine groups in the lateral branches exhibit regions of positive charge density. Azamethiphos (Figure 2C) displays a pronounced negative charge density over the carbonyl oxygen of the ring and the phosphoryl oxygen (P=O), along with a slight positive charge density on the ring carbons adjacent to the chlorine atom. Glyphosate (Figure 2D) shows strong negative charge densities over the oxygens of the carboxylic acid and phosphate groups, and high positive charge densities on the hydrogen atoms of the terminal –OH groups (phosphate and carboxylic acid). Finally, the PET monomer also exhibits negative charge density localized over the carbonyl oxygens of the ester groups.

After evaluating the charge density distribution of the individual molecules, additional analyses were performed to investigate possible interactions between the species. All molecules were evaluated in combination with PFOS. Supplemental Figure S1 presents the electrostatic potential maps for all assessed interactions.

**Figure 3:**
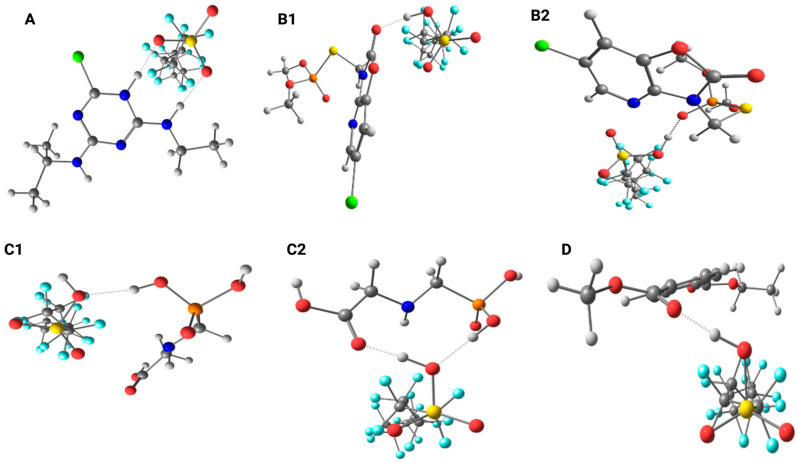
Optimized structures considering the interaction between PFOS and (**A**) atrazine, (**B1**) azamethiphos—carbonyl group, (**B2**) azamethiphos—phosphate group, (**C1**) glyphosate—phosphate group, (**C2**) glyphosate—simultaneous phosphate and carboxyl interactions, and (**D**) PET monomer. Color code: light blue = fluorine; gray = carbon; yellow = sulfur; red = oxygen; white = hydrogen; dark blue = nitrogen; orange = phosphorus; green = chlorine.

The possible molecular interactions can be better visualized in Figure 3. The interaction between PFOS and atrazine (Figure 3A) showed that the charge densities on the chlorine atom and on the atoms of the triazine ring became more positive in the presence of PFOS, probable due to the influence of the fluorine atoms. The calculations indicated that the proximity between the molecules may favor the formation of a hydrogen bond between the –S=O group of PFOS and the nitrogen atom of the –NHCH_2_CH_3_ amine group. Furthermore, the data suggest proton donation from the –SOH group of PFOS to the nitrogen atom of the atrazine ring, followed by the formation of an additional hydrogen bond.

In the evaluation of the interactions between PFOS and azamethiphos, and between PFOS and glyphosate, the data suggests two possible sites for hydrogen bond formation in these herbicides. For azamethiphos, possible interactions can occur at the carbonyl group of the ring (Figure 3B1) and at the phosphate group (Figure 3B2). In both cases, the hydrogen atom from the –SOH group of PFOS acts as the donor in the hydrogen bonding interaction with azamethiphos. In the case of glyphosate, the results indicate the possible formation of a hydrogen bond between the phosphate group of glyphosate and the –SOH group of PFOS (Figure 3C1). Additionally, the calculations showed the potential for simultaneous interactions, in which the same –SOH group of PFOS can form one hydrogen bond with the phosphate group and another with the carbonyl oxygen of the carboxylic acid group in glyphosate (Figure 3C2). Finally, for the PET monomer (Figure 3D), the results indicate a potential interaction between the oxygen atom of the C=O group in the PET ester and the –SOH group of PFOS.

All molecules exhibit regions of positive and negative charge, as well as oxygen atoms that can facilitate intermolecular interactions in solution. These interactions can modify the behavior of these molecules on the environment. Based on the obtained data, it can be suggested that, in the presence of metal ions, these molecules may further interact through electrostatic attraction, forming ion–permanent dipole interactions and possibly metal complexes, owing to the availability of lone electron pairs on the oxygen atoms.

## 4. Conclusions

The coexistence of PFAS and other contaminants in environmental matrices is a reality, raising concerns about ecological and health risks. In vivo and in vitro studies in several aquatic organisms indicate that co-exposure can exacerbate toxicity, leading to a higher incidence of malformations, inhibition of hatching, increased oxidative stress, and alterations in metabolism and gene expression. However, some studies also point to antagonistic interactions, where the presence of one contaminant can mitigate the effects of the other, highlighting the complexity of these interactions. Notably, research on the combined effects of PFAS and pesticides is particularly scarce, despite evidence of synergism observed in aquatic organisms. Similarly, although the ubiquitous presence of microplastics and PFAS in aquatic ecosystems is concerning, studies investigating their combined effects are still in their infancy. The interaction of PFAS with metal ions demonstrates the potential to alter bioavailability and exacerbate toxic effects. Thus, the complexity of interactions between PFAS and other environmental contaminants highlights the urgent need for further research.

This review highlights a significant gap in our understanding of how PFAS interact with other common aquatic contaminants, and how these compounds can influence toxicity in aquatic organisms. Although numerous studies have examined the individual effects of these contaminants, research on their combined effects, particularly at the sub-lethal levels, and studies reporting on synergistic or antagonistic interactions, remain limited. PFAS chemicals can indeed interact with other chemicals, which can lead to differences in availability and receptor binding affinities, as well as novel mechanisms of toxicity. One limitation of our computational approach is that it focuses on chemical interaction only and does not consider the biological activity of such interactions with receptors. Another limitation is that we consider a single PFAS, specifically PFOS, and other PFAS chemicals are expected to show variation in their chemical interactions due to carbon length and unique chemical properties.

A methodological challenge in mixture toxicology studies is replicating environmentally realistic exposures, as aquatic organisms are naturally exposed to dynamic mixtures rather than more precise laboratory conditions. To address this, future research should prioritize environmentally relevant exposure models using field-derived mixtures and chronic, low-dose conditions. Additionally, research should prioritize addressing the scarcity on chronic exposures and multi-trophic level interactions, which remain critical gaps in understanding these complex systems. Efforts should focus on elucidating the mechanisms underlying these complex interactions, investigating effects at different trophic levels and in a broader range of species, including mammalian models, and considering chronic exposures and environmentally relevant mixtures. A more comprehensive understanding of cumulative and long-term exposure effects is essential for improving environmental and human health risk assessments, as well as for developing effective management and remediation strategies to protect the health of aquatic ecosystems and public health.

## Figures and Tables

**Table 1:** Toxicity studies related to the toxicity of PFAS and pesticides, microplastics, or metals in different organisms.

Species	Chemical + Dose	Duration	Effects	Reference
Zebrafish (*Danio rerio*)	5–800 mg/L PFOA 5–15 mg/L atrazine	5 days	Malformations (yolk sac abnormalities, liver abnormalities, spinal curvature)	[54]
Zebrafish (*Danio rerio*)	0.3–30 µmol/L PFOS 10–300 µmol/L cyproconazole 0.3–300 µmol/L triadimefon	5 days	Craniofacial malformations	[41]
Zebrafish (*Danio rerio*)	0.62/10, 6.2/10, 62/10, 620/10 μg/L chlorpyrifos/PFHxA	7 days	Survival and locomotor activity reduced with 620/10 μg/L. Upregulation of neurotoxicity and oxidative stress genes. Reduced ROS.	[55]
Zebrafish (*Danio rerio*)	0.03 mg/L PFOS + 0.1–10 MoS_2_ 0.03 mg/L PFOA + 0.1–10 MoS_2_	2 weeks	Increased bioaccumulation and oxidative stress within liver and intestines	[59]
Cod (*Godus morhua*)	0.1 and 1 µmol/L PFOA 0.1 µmol/L chlorpyrifos 0.01 µmol/L EE2	48 h	Alteration of *cyp24a1* (vitamin-D metabolism), *cyp3a* (xenobiotic metabolism), and *fabp* and *hmgCoA* (lipid/cholesterol metabolism)	[56]
Goldfish (*Carassius auratus*)	1.21 and 12.10 µmol/L PFOA 1 and 10 µmol/L PFOS 0.79 and 3.15 µmol/L Cu	4 days	Decreased CAT and SOD activities	[60]
Seabass (*Dicentrarchus labrax L.*)	4.38 µg/Kg PFOS and 100 mg/Kg MPs	21 days	Lower toxicological alterations of MPs-PFOS, downregulation of immune-related genes, increased bactericidal activity	[61]
Clam (*Scrobicularia plana*)	55.7 µg/g and 46.1 µg/g PFOS and 1 mg/L MPs	14 days	Increased oxidative stress parameters	[62]
Algae (*Chlorella sorokiniana*)	0.05, 0.5, 5 mg/L PFOA and 10 mg/L MPs	96 h	Photosynthesis inhibition, physical damage, and oxidative stress	[63]
Cyanobacteria (*Microcystis aeruginosa*)	100 ng/L–100 mg/L PFOA and 50 mg/L PVC	15 days	Growth inhibition and promotion of synthesis and release of Microcystin-LR	[64]
Cyanobacterium *Anabaena* CPB4337	0–200 mg/L PFOA/PFOS 0–60 mg/L 2,4-D 0–0.75 mg/L atrazine 0–0.05 mg/L diuron 0–0.05 mg/L paraquat	72 h	PFOA increased the toxicity of all herbicides, except for atrazine. PFOS increased paraquat and diuron toxicity and decreased atrazine toxicity.	[58]
*Limnodrilus hoffmeisteri*	pH values (6.2, 7.0 and 8.0) 0–2.4 mg/L Cd 5, 10, and 20 mg/L PFOS	96 h	Cd/PFOS exposure increases acute toxicity	[65]
Water flea (*Daphnia magna*)	70 ng/L PFOS, 7 ng/L PFOA, and 50 mg/L PET	40–60 days	Delayed sexual maturity, suppressed reproduction, triggered developmental failures, and reduced somatic growth	[66]

## Data Availability

No new data were created or analyzed in this study. Data sharing is not applicable to this article.

## Supplementary Materials

The following supporting information can be downloaded at: https://www.mdpi.com/article/10.3390/toxics14050373/s1, Figure S1: Electrostatic potential map.

## Abbreviations

The following abbreviations are used in this manuscript:

2,4-D	2,4-dichlorophenoxyacetic acid
ATPase	Adenosine triphosphatase
B3LYP	Becke three-parameter Lee–Yang–Parr functional
CAT	Catalase
Cd	Cadmium
CERCLA	Comprehensive Environmental Response, Compensation, and Liability Act
ChEs	Cholinesterases
Cr	Chromium
Cu	Copper
DDT	Dichlorodiphenyltrichloroethane
DEHP	Bis-(2-ethylhexyl)-phthalate
DFT	Density functional theory
DNA	Deoxyribonucleic acid
E2	17β-estradiol
EC50	Half maximal effective concentration
EE2	17α-ethinylestradiol
EPA	Environmental Protection Agency
FDA	Food and Drug Administration
Fe	Iron
GD3	Grimme’s dispersion correction (D3)
GRX9	Glutaredoxin 9
GSH	Glutathione
GST	Glutathione S-transferase
GST3	Glutathione S-transferase 3
Hg	Mercury
hmg-CoA	3-hydroxy-3-methylglutaryl–coenzyme A
IEFPCM	Integral equation formalism polarizable continuum model
MeHg	Methylmercury
MDA	Malondialdehyde
MEP	Molecular electrostatic potential
MPs	Microplastics
MoS_2_	Molybdenum disulfide
Ni	Nickel
NPDWR	National Primary Drinking Water Regulation
OCPs	Organochlorine pesticides
Pb	Lead
PCBs	Polychlorinated biphenyls
PE	Polyethylene
PE-MPs	Polyethylene microplastics
PER	Peroxidase
PET	Polyethylene terephthalate
PFAS	Per- and polyfluoroalkyl substances
PFBS	Perfluorobutanesulfonic acid
PFC	Perfluorinated compound
PFHpA	Perfluoroheptanoic acid
PFHxA	Perfluorohexanoic acid
PFHxS	Perfluorohexanesulfonic acid
PFOA	Perfluorooctanoic acid
PFOS	Perfluorooctane sulfonic acid
PFOSA	Perfluorooctanesulfonamide
PFTeDA	Perfluorotetradecanoic acid
PFUnDA	Perfluoroundecanoic acid
PP	Polypropylene
PPCPs	Pharmaceutical and personal care products
PS-MPs	Polystyrene microplastics
PVC	Polyvinyl chloride
*p,p′*-DDE	*p,p′*-dichlorodiphenylethylene
ROS	Reactive oxygen species
SOD	Superoxide dismutase
TMDCs	Transition metal dichalcogenides
TSOD	Total superoxide dismutase
WS_2_	Tungsten disulfide
Zn	Zinc
