# Supplementary material for: Do Perfluorinated Chemicals Enhance the Toxicity of Other Contaminants in Aquatic Organisms? A Review
Source: Toxics. 2026 Apr 26;14(5):373. doi: 10.3390/toxics14050373 (PMC13211294; doi:10.3390/toxics14050373)
Supplement: Supplementary file 1 [file toxics-14-00373-s001.zip › toxics-4204128-supplementary.pdf]

Supplemental Figure

Do perfluorinated chemicals enhance the toxicity of other contaminants in aquatic organisms? A review.

Eliana Maira Agostini Valle[1,2], Emma Ivantsova[1], Maria Luisa Pracchia[2], Calvin Quessada Cabello[3], Hueder Paulo Moisés de Oliveira[3], Lucia Codognoto[2], Christopher J. Martyniuk[1,4*]

[1]Center for Environmental and Human Toxicology, Department of Physiological Sciences, College of Veterinary Medicine, University of Florida, Gainesville, Florida, 32611, USA

[2]Universidade Federal de São Paulo – Instituto de Ciências Ambientais, Químicas e Farmacêuticas – Campus Diadema – Brazil

[3]Centro de Ciências Naturais e Humanas (CCNH), Universidade Federal do ABC, Santo André, Brazil

[4]UF Genetics Institute, Interdisciplinary Program in Biomedical Sciences Neuroscience

*Corresponding author: Christopher J. Martyniuk

email: cmartyn@ufl.edu

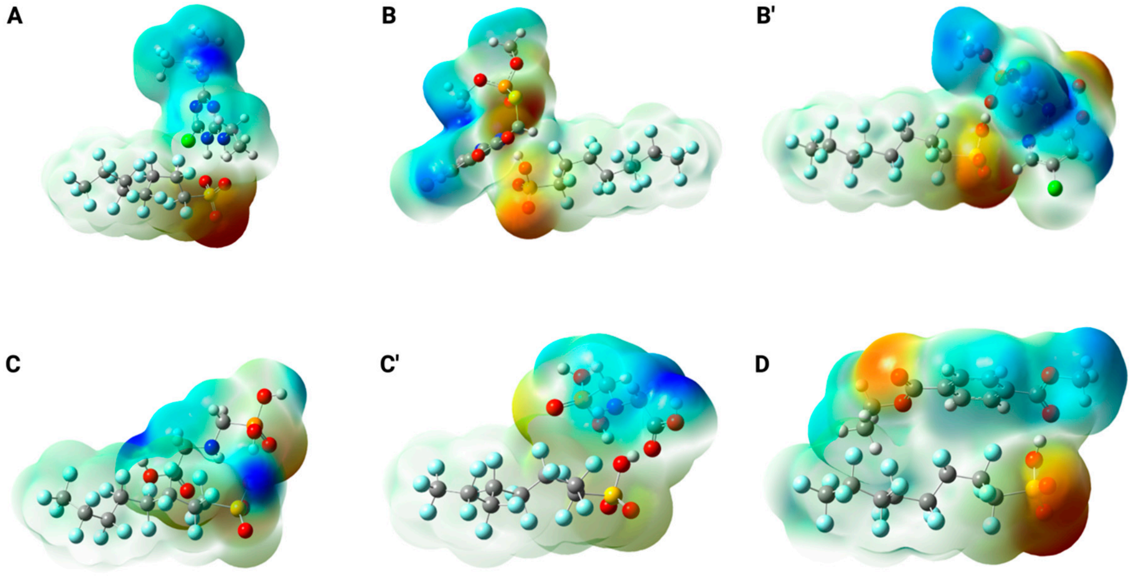

Supplementary Figure S1. Electrostatic Potential Map for the interaction between PFOS and (A) atrazine, (B) azamethiphos – carbonyl group, (B') azamethiphos – phosphate group, (C) glyphosate – phosphate group, (C') glyphosate - simultaneous phosphate and carboxyl interactions and (D) PET monomer. Regions in red indicate high density of negative charge and regions in blue indicate high density of positive charge.
